# Expression Profiling and Clinical Significance of Plasma MicroRNAs in Diabetic Nephropathy

**DOI:** 10.1155/2019/5204394

**Published:** 2019-05-14

**Authors:** Jianqin Wang, Gouqin Wang, Yaojun Liang, Xiaochun Zhou

**Affiliations:** Department of Nephropathy, Lanzhou University Second Hospital, No. 82 Cuiyingmen Lanzhou, Gansu province, China

## Abstract

**Aims:**

MicroRNAs (miRNAs) stably and abundantly exist in body fluids and have been considered as novel and noninvasive biomarkers for several diseases. The present study is aimed at investigating the expression profiling and clinical significance of plasma miRNAs in the pathogenesis and progression of diabetic nephropathy (DN).

**Methods:**

Plasma samples were obtained from 66 DN patients (36 had microalbuminuria and 30 had macroalbuminuria), 36 diabetic patients with normoalbuminuria, and 40 healthy controls. The plasma miRNA profiles were obtained by miRNA low-density array chip and validated by quantitative real-time polymerase chain reaction. The correlations between the differential expression of plasma miRNAs and clinicopathological parameters were explored.

**Results:**

miR-150-5p, miR-155-5p, miR-30e, miR-320e, and miR-3196 were found to be differentially expressed in plasma samples among these three groups: diabetic patients with microalbuminuria, diabetic patients with normoalbuminuria, and healthy controls (*P* < 0.05). The expression levels of miR-150-5p and miR-155-5p in patients with macroalbuminuria were 2.3-fold (*P* = 0.001) and 1.5-fold (*P* = 0.033) higher than patients with microalbuminuria, respectively. However, the expression levels of miR-30e, miR-3196, miR-320, and let-7a-5p were not significantly different between these two groups (*P* > 0.05). Furthermore, plasma miR-150-5p (*P* = 0.016, *r* = -0.460) and miR-155-5p (*P* = 0.014, *r* = -0.467) were negatively correlated with the albuminuria excretion rate, while plasma miR-150-5p (*P* = 0.01, *r* = 0.318) and miR-155-5p (*P* = 0.030, *r* = 0.271) were positively correlated with the estimated glomerular filtration rate.

**Conclusion:**

miR-150-5p, miR-155-5p, miR-30e, miR-320e, and miR-3196 are potentially new diagnostic biomarkers for early DN. miR-150-5p and miR-155-5p may be involved in the pathogenesis and progression of DN. Further research is required to verify these findings and clarify the specific molecular mechanisms.

## 1. Introduction

Diabetic nephropathy (DN) is one of the most known microvascular complications of diabetes mellitus (DM), which presents with persistent proteinuria, a progressive decrease in glomerular filtration rate, and elevation in blood pressure [1]. The pathogenesis of DN is complex and includes various factors, such as glucose metabolism disorders, renal hemodynamic changes, cytokines, oxidative stress, and genetic susceptibility, which have not yet been completely clarified. Although attempts directed at delaying the progression of DN through strict control of blood glucose and blood pressure have been made at present, these treatments cannot completely stop or reverse the progression of DN [2]. Therefore, more studies are needed to explore and connect the key links between the pathogenesis and development of DN and thereby help to investigate new methods to prevent the occurrence and development of the disease.

Conventionally, the severity of DN is assessed by measuring urinary albumin excretion, such as albumin-to-creatinine ratio (ACR). Microalbuminuria is typically considered as a marker of DN, while persistent macroalbuminuria is regarded as a predictor of ESRD [3, 4]. However, there are conflicts in recent studies regarding the sensitivity and specificity of urinary albumin excretion for DN [5–7]. Thus, a reliable tool is needed to evaluate the initiation and development of DN for early intervention.

MicroRNA (miRNA) is a small noncoding RNA with a length of 21–24 nucleotides, which has been found in plants, animals, and some viruses. Through base-pairing, the miRNA binds to the target messenger RNA (mRNA), leading to cleavage, degradation, or translation of the target mRNA. In recent studies, miRNAs have been found to be secreted by cells into body fluids, and these circulating miRNAs are highly stable. Since the expression profiles of these circulating miRNAs are often associated with specific diseases, such as cancers, these exhibit great potential as noninvasive or minimally invasive biomarkers for the diagnosis of various diseases [8]. Recent evidence has indicated that miRNA signatures as biomarkers and therapeutic targets for DN [8]. Moreover, several studies have suggested that miRNA expression can help in understanding the complex pathogenesis of DN and can be used as an important “biological tool” to diagnose the initiation and development of DN [8, 9].

Considering the potential role of miRNAs in DN, in the present study, the expression profiling and clinical significance of plasma miRNAs in the pathogenesis and progression of DN were investigated.

## 2. Materials and Methods

### 2.1. Participants and Grouping

The present study comprised of 106 consecutive patients diagnosed with type-2 diabetes, who were admitted in Lanzhou University Second Hospital from January to June 2014. In addition, a total of 40 healthy age- and gender-matched participants were included as normal controls (NC group). According to the presence of albuminuria, the diabetic patients were divided into two groups: diabetes with normoalbuminuria, which was defined as a urinary albumin excretion rate (UAER) of <30 mg/24 hours and a serum creatinine (Scr) of <133 *μ*mol/L (DM group, *n* = 40) [10]; diabetes with albuminuria (presence with DN), which was defined as a UAER of >30 mg/24 hours (DN group, *n* = 66). The DN group was further subdivided into two groups: microalbuminuria group, defined as 30 mg/24 hours < UAER of <300 mg/24 hours (Mic group, *n* = 36); macroalbuminuria, defined as a UAER of >300 mg/24 hours (Mac group, *n* = 30). Patients were excluded when they were over 65 years old or had ketoacidosis, malignancy, trauma, infection, hepatitis, human immunodeficiency virus (HIV) positivity, or autoimmune disease. The study protocol was approved by the Ethics Committee of Lanzhou University Second Hospital, and an informed consent was obtained from all participants. These patients were divided as follows: screening set (10 patients in the DM, Mic, and NC group, respectively) and validation set (40 patients in the DM group, 36 patients in the Mic group, and 40 patients in the NC group).

### 2.2. Blood Processing

Two mL of fasting peripheral venous blood samples was collected using EDTA-coated vacuum tubes. Then, the samples were centrifuged at 800 g for 15 minutes at room temperature within one hour of collection. Afterwards, 1.5 mL of plasma was transferred to Eppendorf tubes and centrifuged at 12,000 g for five minutes. Subsequently, the plasma was transferred to a new 1.5 mL Eppendorf tube and preserved at -80°C.

### 2.3. miRNA Transcriptome Profile

All plasma samples were sent to Shanghai KangChen Bio-tech and analyzed using the miRNA TaqMan low-density array, which contained a total of 325 miRNAs. Briefly, the plasma samples of diabetic patients and healthy controls, which were preserved at -80°C, were fully thawed in room temperature. Then, 200 *μ*L of plasma samples was mixed with the QIAzol Lysis Reagent (QIAGEN, Hilden, Germany) at a 1 : 5 volume for 15 minutes. Afterwards, the RNA was extracted according to the instructions of the miRNeasy Serum/Plasma extraction kit (QIAGEN, Germany), followed by chloroform deproteinization and ethanol precipitation. Subsequently, the RNA was dissolved in 12 *μ*L of diethyl pyrocarbonate- (DEPC, Takara Biotechnology, China) treated water.

After having passed the RNA quantity measurement using the NanoDrop 1000 (Thermo Fisher Scientific, Waltham, MA, USA), the samples were labeled using a miRCURY™ Hy3™/Hy5™ Power labeling kit (Exiqon, Vedbaek, Denmark) and hybridized on the miRCURY™ LNA Array (v.18.0, Exiqon, Denmark). Following the washing steps, the slides were scanned using an Axon GenePix 4000B microarray scanner (Axon Instruments, Foster City, CA, USA).

Next, the scanned images were imported into the GenePix Pro 6.0 software (Axon) for grid alignment and data extraction. Replicated miRNAs were averaged, and miRNAs with intensities ≥30 in all samples were chosen to calculate the normalization factor. The expressed data were normalized using median normalization. After normalization, differentially expressed miRNAs were identified through fold-change filtering. Finally, hierarchical clustering was performed to present the distinguishable miRNA expression profiling among samples.

### 2.4. Quantitative Real-Time Polymerase Chain Reaction (qPCR)

Quantitative real-time PCR with TaqMan probes was used to validate the reproducibility of the results from the miRNA microarray. The total RNA (10–30 *μ*g) was reverse-transcribed into the first-strand cDNA which was performed using the One Step PrimeScript miRNA cDNA Synthesis kit (Takara Bio Inc., Shiga, Japan) and a universal oligo(dT)-containing adaptor primer. The reaction conditions of the PCR amplification were as follows: 37°C for 60 minutes of miRNA poly 3′polyadenylation for the reverse transcription of the cDNA and 85°C for five seconds for DNA polymerase inactivation. These were performed on an ABI 7500 fluorescence quantitative PCR instrument (Applied Biosystems, Foster City, CA, USA) using a SYBR Green fluorescent quantitative PCR kit (Takara, Japan). The cycling condition included two steps: a single initial cycle (95°C for 30 s), followed by 40 cycles (95°C for 5 s, 60°C for 34 s). An exogenous cel-miR-39 (synthetic miRNA from *C. elegans*) was used as a spike-in control for normalization.

### 2.5. Estimated Glomerular Filtration Rate (eGFR)

The eGFR was estimated through the following equation: Chronic Kidney Disease Epidemiology creatinine (CKD-Epi_Cr) equation = 141 × min (Scr/*κ*, 1)*α* × max (Scr/*κ*, 1) − 1.209 × 0.993age × 1.018 (when female) × 1.159 (when black), where Scr was defined as serum creatinine, *κ* was 0.7 for females and 0.9 for males, *α* was −0.329 for females and −0.411 for males, min was the minimum of Scr/*κ* or 1, and max was the maximum of Scr/*κ* or 1 [10].

### 2.6. Statistical Analysis

Statistical analysis was performed using SPSS 19.0. Continuous data were expressed as mean ± standard deviation (x ± SD), and categorical data were expressed as an absolute value and percentage. The comparisons of continuous variables between these groups were performed using Student's *t*-tests for normally distributed data and the Mann-Whitney *U*-test for nonnormally distributed data. Data expressed in terms of proportions were compared using the chi-square test or Fisher's exact test. One-way ANOVA followed by the Student–Newman–Keuls analysis was used to determine the significance of the differences in the miRNA levels between groups. The relationship between miRNAs and clinical parameters was assessed using the bivariate correlation method (Spearman's rank order correlation). Receiver operating characteristic (ROC) curves were constructed to evaluate the diagnostic value of miRNAs. The area under the curve (AUC) and 95% confidence intervals (CI) were calculated to determine the specificity and sensitivity. The ROC curves and multivariate logistic regression in the ROC curves were calculated using the SigmaPlot 12.5 software. Statistical significance was defined as a *P* value of < 0.05.

## 3. Results

### 3.1. Screening of Plasma miRNAs

TaqMan low-density array chip technology was used to detect the pooled plasma samples of 10 randomly selected cases from each group (Mic, DM, and NC groups). A significant difference in the miRNA expression was defined as a ≥2-fold increase or decrease and Ct values of <30. Compared with the NC group, the Mic group exhibited an increased expression in 158 miRNAs and a decreased expression in 150 miRNAs. Compared with the DM group, 128 miRNAs had an increased expression and 95 miRNAs had a decreased expression in the Mic group.

**Figure 1:**
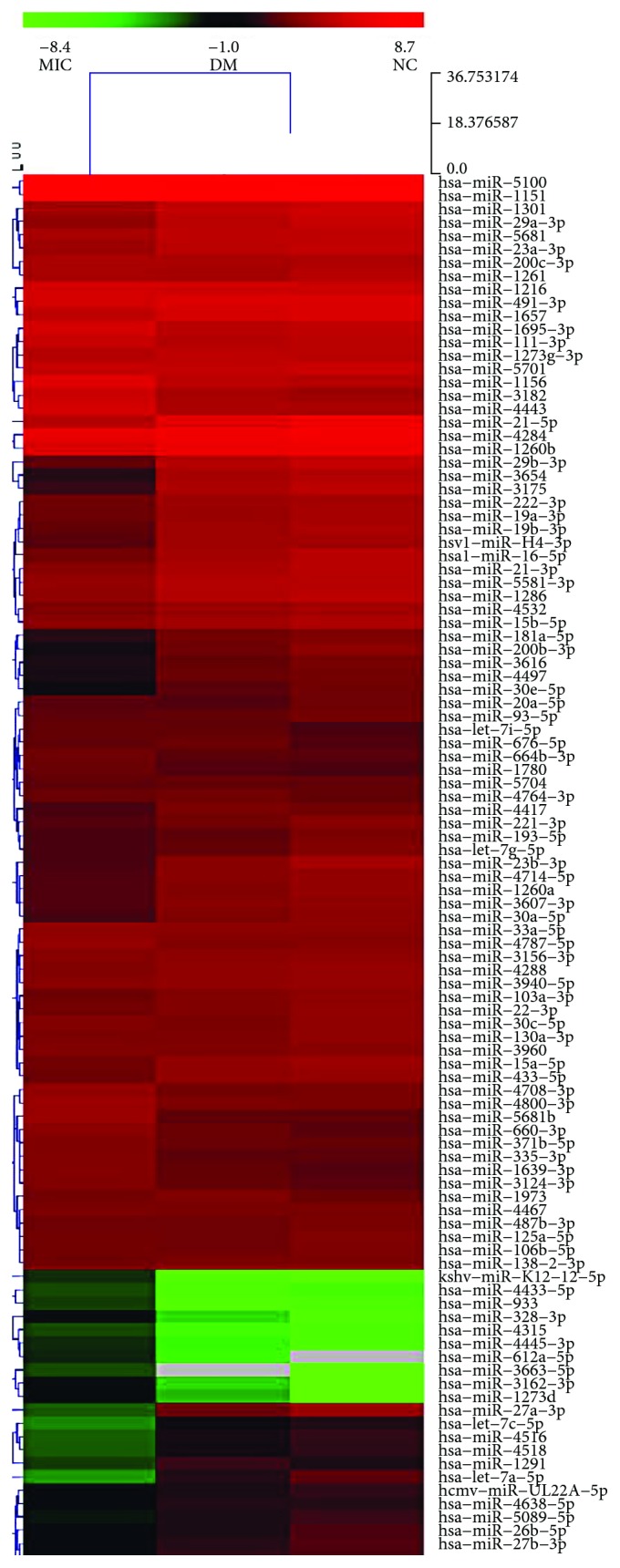
Plasma miRNA microarray expression profile (part). RNA was extracted from plasma samples obtained from diabetic patients with microalbuminuria (Mic, *n* = 10), diabetes mellitus patients with normoalbuminuria (DM, *n* = 10), and normal controls (NC, *n* = 10).

The results obtained from the chips were integrated into an online software (e.g., TargetScan, PicTar, or miRanda) for target gene prediction and differentially expressed miRNAs, which were defined as a ≥2-fold increase or decrease in all three groups, were identified, including 20 identified miRNAs: miR-27a-3p, miR-30e, miR-33b, miR-50, miR-125b-5p, miR-150-5p, miR-155-5p, miR-296, miR-320e, miR-328, miR-484, miR-487, miR-550a-5p, miR-590-5p, miR-744, miR-885-5p, miR-933, miR-3196, let-7a-5p, and let-7c-5p. The results of the chip analysis for examining the plasma miRNA expression profiles are presented in Figure 1 (the data of the complete graph of the array chip is presented in Supplementary Materials (available here)).

### 3.2. Validation and Analysis of the Gene Chip

The results of the chip analysis were validated by quantitative PCR in another set of participants, including the NC (*n* = 40), DM (*n* = 40), and Mic (*n* = 36) groups. The clinical characteristics of patients in these three groups are presented in Table 1.

**Figure 2:**
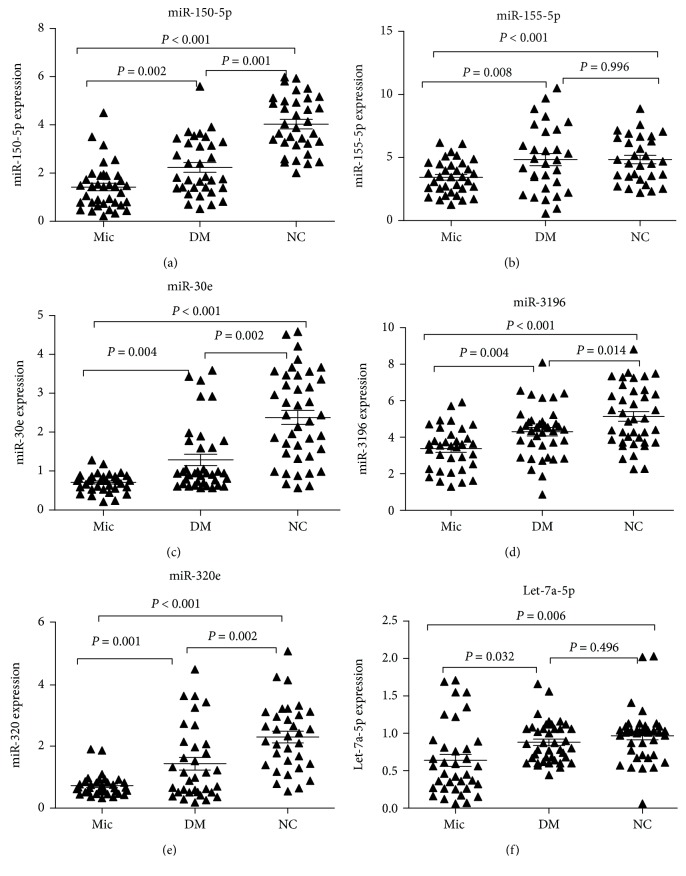
Analysis of the relative transcription levels of plasma miRNAs using the nonparametric Mann-Whitney *U*-test. The expression of miR-155-5p (a), miR-150-5p (b), miR-30e (c), miR-3196 (d), miR-320e (e), and let-7a-5p (f) was determined by quantitative real-time PCR. *P* < 0.05 and *P* < 0.001 indicate statistical significance and highly statistical significance, respectively. Mic: diabetes mellitus with microalbuminuria (*n* = 36); DM: diabetes mellitus with normoalbuminuria (*n* = 40); NC: normal controls (*n* = 40). The error bars are presented as standard error of the mean (SEM).

The quantitative PCR analysis revealed that the expression of plasma miR-125b-5p (*F* = 19.812, *P* < 0.001), miR-484 (*F* = 6.489, *P* = 0.003), and miR-550 (*F* = 3.652, *P* = 0.034) increased, while the expression of miR-30e (*F* = 17.313, *P* < 0.001), miR-155-5p (*F* = 6.369, *P* = 0.002), miR-320 (*F* = 12.309, *P* < 0.001), let-7a-5p (*F* = 8.034, *P* = 0.002), miR-150-5p (*F* = 37.733, *P* < 0.001), and miR-3196 (*F* = 6.372, *P* = 0.002) decreased in the Mic group, when compared with the NC and DM groups (Table 2 and Figure 2). A total of 10 miRNAs were statistically significant among these three groups.

There were no significant differences in the expression levels of miR-125b-5p, miR-484, miR-550, and let-7c-5p between the Mic and DM groups (*P* > 0.05). However, significant differences were found in miR-155-5p, miR-150-5p, miR-30e, miR-3196, miR-320e, and let-7a-5p expression between these two groups (*P* < 0.05).

### 3.3. Diagnostic Value of Plasma miRNAs in DN

**Figure 3:**
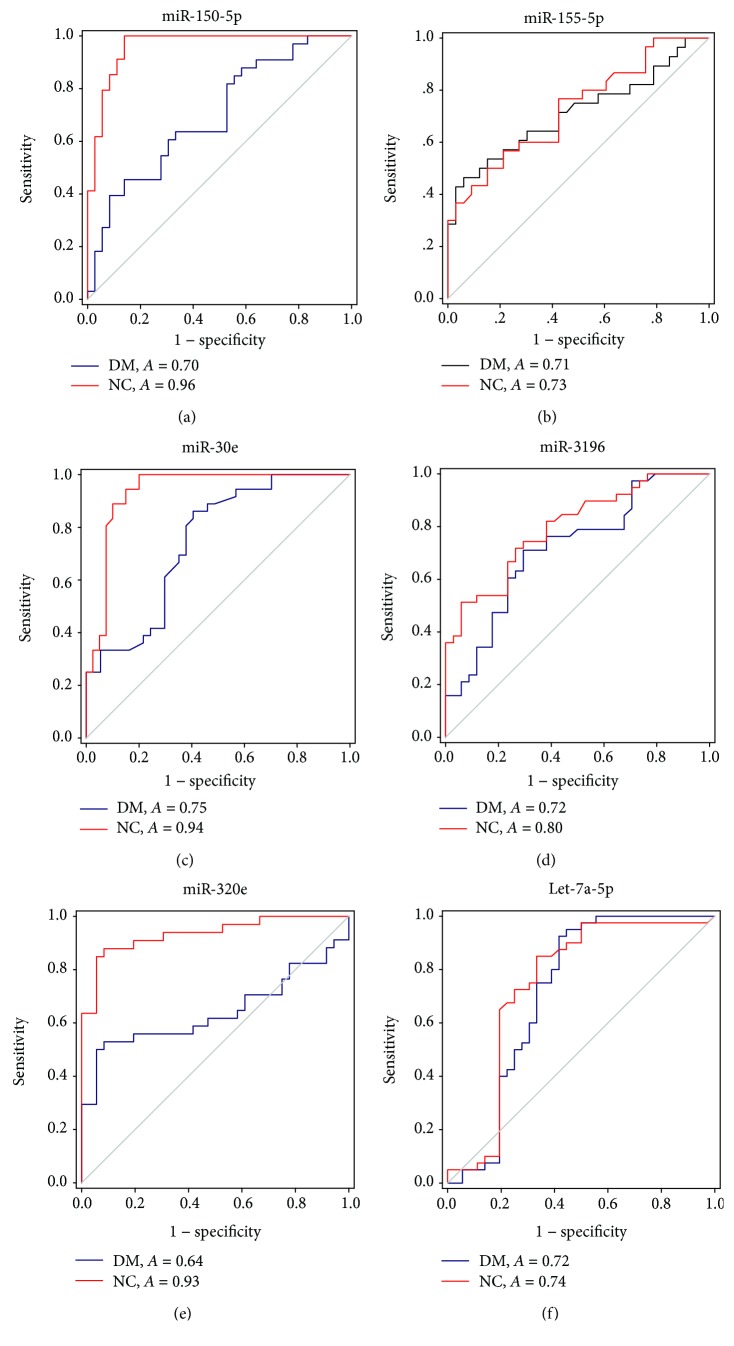
The ROC curve was constructed to evaluate the diagnostic performance of plasma miRNAs: (a) miR-155-5p, (b) miR-150-5p, (c) miR-30e, (d) miR-3196, (e) miR-320e, and (f) let-7a-5p. The red lines represent the ROC curves for differentiating DN from NC, while the blue lines represent the ROC curves for differentiating DN from NC.

ROC curves were constructed to evaluate the diagnostic value of plasma miR-150-5p, miR-155-5p, miR-30e, miR-3196, miR-320e, and let-7a-5p for DN (Figure 3). The diagnostic performance of plasma miRNAs to differentiate DN from DM or NC was summarized in Table 3. Plasma miR-150-5p, miR-155-5p, miR-30e, miR-3196, and let-7a-5p could differentiate DN from NC, as well as DM, with both AUC > 0.7, indicating a potential diagnostic value for DN.

### 3.4. Relative Expression Levels of Plasma miR-155-5p, miR-150-5p, miR-30e, miR-3196, miR-320e, and let-7a-5p in the DN Group

**Figure 4:**
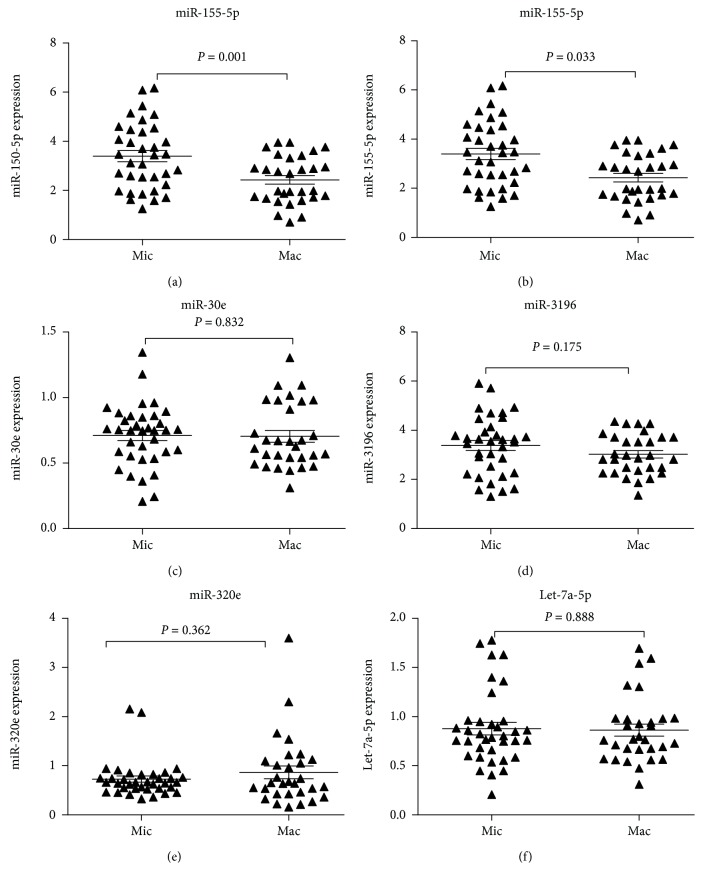
Comparison of the expression levels of miRNAs between the Mac and Mic groups. The miRNA expression levels were determined by quantitative real-time PCR. The expression of miR-150-5p (a) and miR-155-5p (b) was approximately 2.3-fold (*P* = 0.001) and 1.5-fold (*P* = 0.033) higher in the Mic group, when compared to the Mac group, respectively. There were no significant differences in the expression levels of miR-30e (c), miR-3196 (d), miR-320e (e), and let-7a (f) between these two groups. Mic: microalbuminuria (*n* = 36); Mac: macroalbuminuria (*n* = 30). The error bars are presented as standard error of the mean (SEM).

There were no differences in the expression levels of plasma miR-30e (*P* = 0.832), miR-3196 (*P* = 0.175), miR-320e (*P* = 0.362), and let-7a-5p (*P* = 0.888) between the Mac and Mic groups (*P* > 0.05) (Figure 4). However, the expression of miR-150-5p and miR-155-5p was approximately 2.3-fold (*P* = 0.001) and 1.5-fold (*P* = 0.033) higher in the Mic group, when compared to the Mac group, respectively.

### 3.5. Correlations between Plasma miR-155-5p, miR-150-5p, and Clinical Parameters in DN Patients

There were significant differences in blood pressure, fasting plasma glucose, high-density lipoprotein, Scr, blood urea nitrogen, eGFR, UAER, the presence of retinopathy, and jugular vein intima-media thickness between the DN and DM groups (*P* < 0.05, Table 1). However, no significant differences were found in these two groups in terms of age, gender, duration of diabetes, body mass index, triglycerides, low-density lipoprotein, and total cholesterol (all, *P* > 0.05).

**Figure 5:**
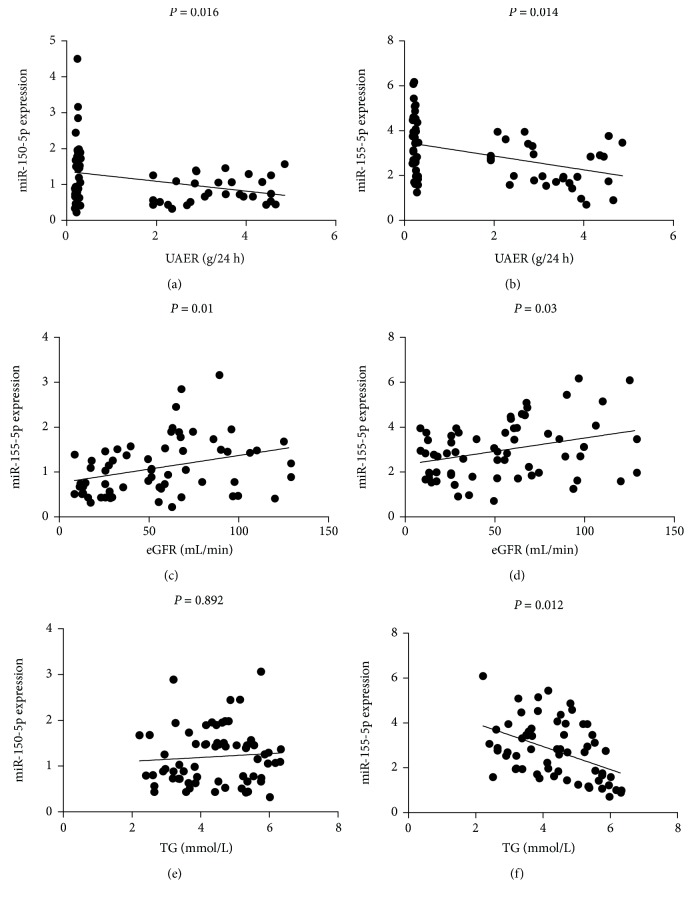
The correlation of plasma miR-150-5p and miR-155-5p expression levels with clinical parameters in patients with DN. Plasma miR-150-5p (*P* = 0.016, *r* = −0.460; (a)) and miR-155-5p (*P* = 0.014, *r* = −0.467; (b)) were negatively correlated with UAER. Plasma miR-150-5p (*P* = 0.01, *r* = 0.318; (c)) and miR-155-5p (*P* = 0.030, *r* = 0.271; (d)) were positively correlated with eGFR values. (e) No correlation was observed between miR-150-5p and triglycerides (*P* = 0.892, *r* = 0.027). (f) Plasma miR-155-5p expression was positively correlated with triglycerides (*P* = 0.012, *r* = −0.403). UAER: urinary albumin excretion rate; EGFR: estimated glomerular filtration rate; TG: triglycerides.

Further analysis was carried out to evaluate the correlations between plasma miR-155-5p, miR-150, and clinical parameters in DN patients (Figure 5). Plasma miR-150-5p (*P* = 0.016, *r* = −0.460) and miR-155-5p (*P* = 0.014, *r* = −0.467) were negatively correlated with the albuminuria excretion rate. On the contrary, plasma miR-150-5p (*P* = 0.01, *r* = 0.318) and miR-155-5p (*P* = 0.030, *r* = 0.271) were positively correlated with eGFR values. Furthermore, plasma miR-155-5p expression had a negative correlation with triglycerides (*P* = 0.012, *r* = −0.403), while no such correlation was observed between miR-150-5p and triglycerides (*P* = 0.892, *r* = 0.027).

## 4. Discussion

Although the present diagnosis and monitoring of DN heavily rely on the detection of urinary microalbuminuria, it is not sensitive, because tissue damage and inflammation have already occurred by the time that microalbuminuria is detectable. Furthermore, microalbuminuria is not specific to DN but is merely a hallmark of glomerular lesions. Renal biopsy is the gold standard for diagnosing DN, but it is a highly invasive and expensive procedure, with a potential risk of biopsy-associated bleeding complications. Thus, sensitive and reliable biomarkers are needed for DN. miRNAs are small molecules of noncoding RNA and are important in a variety of physiological and pathological processes in the body, such as the regulation of renal response to hyperglycemia, and the process of chronic renal disease, including DN. The specific expression profile can be found in the plasma/serum of patients, and changes in expression levels can reflect the condition of patients to a certain extent [11, 12]. Klimczak et al. reported that plasma microRNA-155-5p is elevated in patients with chronic kidney disease and nocturnal hypertension [13]. In addition, several miRNAs, such as plasma miRNA-130b, and their significant correlation with DN have been reported [14–16]. In addition, Zhang et al. reported the use of urinary miR-196a, miR-30a-5p, and miR-490 as novel noninvasive biomarkers for evaluating the activity of focal segmental glomerulosclerosis [17]. In the present study, the expression profiling and clinical significance of plasma miRNA in the course of DN were investigated. Plasma miRNA levels were compared among diabetic patients with normoalbuminuria and microalbuminuria, as well as healthy controls. Compared with diabetic patients with normoalbuminuria and controls, plasma miR-155-5p, miR-150-5p, miR-30e, miR-3196, miR-320e, and let-7a-5p levels were significantly lower in diabetic patients with microalbuminuria, suggesting the decreased plasma levels of these miRNAs at the early stage of DN. Furthermore, miR-155-5p, miR-150-5p, miR-30e, miR-3196, and let-7a-5p achieved an AUC of >0.7 for diagnosing DN, when differentiated from diabetes patients and healthy controls, suggesting a potential diagnostic biomarker of early DN.

Recent studies have confirmed that the miR-30 family is involved in the injury of podocytes, glomerulosclerosis, and proteinuria [18]. Furthermore, the downregulation of miR-30e is associated with TGF-*β*1-mediated epithelial–mesenchymal transition (EMT) and kidney fibrosis [19], while the overexpression of miR-30e could promote the proliferation of renal tubular epithelial cells and inhibit EMT, ultimately preventing renal fibrosis in DN [20]. Present studies on miR-3196 have been predominantly focused on tumors, and there are no reports related to kidney diseases [21, 22]. The miRNA let-7 family is involved in cell proliferation and differentiation [23]. The decrease in their expression is implicated in the pathological changes of EMT, which enhances the ability of cell migration and invasion [24, 25]. In the study conducted by Brennan et al., the let-7 family was found to have a protective effect on kidneys, and the decreased expression of let-7 was relevant to the pathogenesis of fibrotic change [26]. Furthermore, it was found that the plasma let-7 level significantly declined in patients with diabetes and that this could increase again after glycemic control improved [27]. Zhou et al. found that the polymorphism of let-7a was closely correlated with the increased risk for DN [28]. Furthermore, miR-320 has been reported to be involved in the regulation of ischemic-reperfusion-induced cardiac injury and dysfunction [29]. The decreased expression of miR-320 was found in the plasma of patients with diabetes [30]. In addition, high glucose exposure decreased the expression of miR-320 in human umbilical vein endothelial cells and kidney tissues of diabetic rats [31]. In the present study, it was found that among diabetes patients, plasma miR-30e, miR-320e, miR-3196, and let-7a levels decreased at the early stage of DN. However, no significant differences were found in these four miRNAs between diabetic patients with microalbuminuria and macroalbuminuria. Taken together, these results may suggest that miR-30e, miR-3196, miR-320e, and let-7a are mainly involved in early DN. Further research is needed to confirm the detailed molecular mechanism.

The differential expression of the six selected plasma miRNAs was further compared between patients with microalbuminuria and macroalbuminuria. The results revealed miR-150-5p and miR-155-5p levels further decreased in the macroalbuminuria group, when compared with the microalbuminuria group. However, no significant differences were detected in the expression levels of miR-30e, miR-3196, miR-320e, and let-7a between these two groups. These results suggest the possible association between miR-150-5p, miR-155-5p, and the development of DN. Moreover, plasma miR-155-5p and miR-150-5p levels in patients with DN were positively correlated with eGFR but were negatively correlated with the urinary protein excretion rate, suggesting that the decrease in the miR-155-5p and miR-150-5p levels may be associated with the development of renal impairment.

Recent studies have shown that the abnormal expression of miR-155 is correlated to tumors and autoimmune and cardiovascular diseases [32, 33]. A study compared the expression levels of miR-155 in serum samples obtained from diabetic and healthy subjects. The results revealed that the reduced level of miR-155 was associated with obesity and its related metabolic traits, such as hyperinsulinemia and dyslipidemia [34]. Furthermore, the miR-155 expression was found to be elevated in renal tissue of patients with DN, which was mainly located in the glomerular endothelial, mesangial, and tubular cells [35]. With the progression of the disease, the abundance of miR-155 has become even higher, revealing the involvement of miR-155 in the development of DN, which was probably due to its response to the reaction of kidney inflammation. In addition, this study found a close correlation between miR-155 and the level of serum creatinine, indicating miR-155 as a good biomarker of kidney injury [35]. Studies on miR-150 have also mainly focused on lymphocyte-associated tumors and autoimmune diseases [36, 37]. A high concentration of circulating miR-150 could suppress the growth of malignant tumors, reduce collagen synthesis, and enhance the migration of microvascular endothelial cells in humans [38]. A low concentration of circulating miR-150 is correlated to ventricular remodeling after myocardial infarction, pulmonary hypertension, and the poor recovery of sepsis patients [39–41]. In a study that examined the expression of miR-150 in kidney biopsies obtained from patients with lupus nephritis, miR-150 was found to promote renal fibrosis, which was probably induced by increasing profibrotic molecules through the downregulation of SOCS1 [42]. In the present study, miR-150 and miR-155-5p exhibited a persistent declining trend irrespective of the early and progressive stages of DN. It was speculated that this was probably induced through some complex mechanisms to reduce the release of microRNAs from tissues into circulation during DN, but the specific mechanism remains unclear. Thus, the exact mechanism by which miR-150 and miR-155-5p works in the pathological process of DN would be studied in future research.

The present study had a few limitations. First, this was a single center retrospective cohort study with a relatively small sample size. Second, the tissue expression levels of miRNAs in kidneys were not detected, which needs further investigation.

In conclusion, the expression levels of plasma miR-150-5p, miR-155-5p, miR-30e, miR-320e, and miR-3196 decreased at the early stage of DN, suggesting that these can be used as new diagnostic biomarkers for early DN. Furthermore, miR-150-5p and miR-155-5p further decreased during the progression of DN and correlated with eGFR and UAER, which are two clinical parameters that reflect the severity of renal impairment. These suggest the involvement of these two miRNAs in the pathogenesis and progression of DN. Further research is required to clarify these specific molecular mechanisms, allowing therapies and other interventions to be created accordingly.

## Figures and Tables

**Table 1:** Characteristics of participants.

Parameters	NC group (*n* = 40)	DM group (*n* = 40)	DN group (*n* = 66)	DN group	
				Mic group (*n* = 36)	Mac group (*n* = 30)
Gender (male/female)	21/19	22/18	34/32	20/16	14/16
Age (years)	55.12 ± 4.99	56.72 ± 6.07	57.22 ± 5.66	56.82 ± 5.53	57.52 ± 5.87
Duration of diabetes (years)	—	8.61 ± 3.28	8.87 ± 2.40	8.82 ± 1.84	8.82 ± 2.74
FPG (mmol/L)	4.71 ± 0.60	7.46 ± 2.28	8.02 ± 2.55	7.72 ± 2.57	8.62 ± 2.67
Blood urea nitrogen (mmol/L)	4.63 ± 0.87	4.83 ± 0.95	9.12 ± 4.04^∗#^	6.04 ± 0.95	12.22 ± 3.43^a^
Serum creatinine (*μ*mol/L)	62.96 ± 12.42	68.02 ± 11.12	188.01 ± 198.42^∗#^	63.33 ± 14.54	312.82 ± 219.25^a^
Triglyceride (mmol/L)	4.14 ± 0.82	4.72 ± 1.37	4.23 ± 1.15	3.94 ± 0.91	4.54 ± 1.60
Total cholesterol (mmol/L)	1.65 ± 0.75	2.63 ± 1.91	2.33 ± 1.35	2.47 ± 1.63	2.13 ± 1.06
LDL-C (mmol/L)	1.74 ± 0.65	2.92 ± 0.70	2.66 ± 1.13^#^	2.65 ± 0.82	2.61 ± 1.38
HDL-C (mmol/L)	0.91 ± 0.32	0.9 ± 0.22	1.13 ± 0.41^∗^	1.05 ± 0.22	1.21 ± 0.46
Retinopathy, *n* (%)	0 (0.0)	16 (40.0)	49 (74.2)^∗^^#^	23 (63.9)	26 (86.7)^a^
cIMT (cm)	—	0.10 ± 0.01	0.13 ± 0.03^∗^	0.11 ± 0.02	0.16 ± 0.01^a^
Hypertension, *n* (%)	0 (0.0)	15 (37.5)	45 (68.2)^∗^^#^	18 (50.0)	27 (90.0)^a^
Cardiomegaly, *n* (%)	0 (0.0)	19 (47.5)	48 (72.7)^#^^∗^	21 (58.3)	27 (56.7)^a^

NC: normal controls; DM: diabetes mellitus with normoalbuminuria; Mic: diabetic nephropathy with microalbuminuria; Mac: diabetic nephropathy with macroalbuminuria. FPG: fasting plasma glucose; LDL-C: low-density lipoprotein cholesterol; HDL-C: high-density lipoprotein cholesterol; cIMT: carotid intima-media thickness. Comparisons were made between groups using the nonparametric Mann-Whitney *U*-test. ^∗^ *P* < 0.05 between DM vs. DN; ^#^ *P* < 0.05 between NC vs. DN; ^a^ *P* < 0.05 between Mac vs. Mic.

**Table 2:** Expression of plasma miRNAs in the three groups.

miRNAs	Mic (*n* = 36)	DM (*n* = 40)	NC (*n* = 40)	*P* values
miR-27a-3p	0.20 ± 0.20	0.66 ± 0.35	0.97 ± 1.87	0.303
miR-30e	0.60 ± 0.23^#^∗^^	1.16 ± 0.81	2.28 ± 1.25	<0.001
miR-33b	1.20 ± 0.22	0.76 ± 0.32	0.78 ± 0.87	0.821
miR-50	1.23 ± 0.63	1.56 ± 1.06	1.64 ± 1.13	0.854
miR-125b-5p	6.70 ± 4.64^#^	1.77 ± 1.84	0.91 ± 0.75	<0.001
miR-150-5p	1.47 ± 0.87^#^∗^^	2.68 ± 1.85	4.03 ± 1.15	<0.001
miR-155-5p	3.43 ± 1.33^#^∗^^	5.06 ± 2.91	5.49 ± 2.36	0.002
miR-296	1.17 ± 0.78	3.56 ± 2.06	3.64 ± 4.13	0.086
miR-320e	0.65 ± 0.30^#^∗^^	1.56 ± 1.21	2.38 ± 1.89	<0.001
miR-328	1.65 ± 1.80	2.00 ± 1.39	1.96 ± 2.26	0.889
miR-484	6.06 ± 3.55^#^	4.91 ± 1.64	2.51 ± 1.97	0.001
miR-487	0.43 ± 0.30	0.98 ± 0.66	1.47 ± 1.73	0.120
miR-550a-5p	7.51 ± 3.51^#^	6.31 ± 1.41	4.80 ± 1.28	0.005
miR-590-5p	1.07 ± 0.86	0.66 ± 0.54	0.20 ± 0.16	0.099
miR-744	4.78 ± 2.26	4.78 ± 2.27	4.78 ± 2.28	0.836
miR-885-5p	3.34 ± 3.94	1.71 ± 2.05	1.04 ± 0.71	0.146
miR-933	4.21 ± 1.26	5.46 ± 2.32	5.64 ± 1.34	0.842
miR-3196	3.03 ± 1.24^#^∗^^	5.41 ± 2.51	5.91 ± 2.68	0.002
let-7a-5p	0.40 ± 0.37^#^∗^^	0.92 ± 0.36	1.20 ± 0.58	0.001
let-7c-5p	1.46 ± 0.95^#^	3.71 ± 3.59	7.44 ± 4.74	<0.001

NC: normal controls; DM: diabetes mellitus with normoalbuminuria; Mic: diabetic nephropathy with microalbuminuria. Data were presented as mean ± standard deviation (SD). ^∗^ *P* < 0.05 between DM vs. Mic; ^#^ *P* < 0.05 between NC vs. Mic. *P* values were calculated by one-way ANOVA analysis, followed by the Student–Newman–Keuls analysis.

**Table 3:** Diagnostic value of plasma miRNAs for DN.

	Sensitivity (%)	Specificity (%)	AUC	95% CI
Prediction of DN from DM				
miR-150-5p	45.5	86.1	0.70	0.576–0.849
miR-155-5p	46.3	93.9	0.71	0.632–0.859
miR-30e	59.4	86.1	0.75	0.582–0.882
miR-3196	71.1	70.6	0.72	0.597–0.836
miR-320e	52.9	91.7	0.64	0.501–0.782
let-7a-5p	50.0	94.4	0.72	0.588–0.846
Prediction of DN from NC				
miR-150-5p	100	83.3	0.96	0.603–0.853
miR-155-5p	56.7	78.8	0.73	0.603–0.853
miR-30e	85.0	94.4	0.94	0.878–0.995
miR-3196	84.6	77.1	0.80	0.698–0.897
miR-320e	87.9	91.6	0.93	0.875–0.993
let-7a-5p	87.9	91.7	0.74	0.619–0.869

DN: diabetic nephropathy; NC: normal controls; DM: diabetes mellitus with normoalbuminuria.

## Data Availability

The data used to support the findings of this study are available from the corresponding author upon request.

## Supplementary Materials

Supplementary Materials Supplementary Figure 1: the complete graph of the array chip. RNA was extracted from plasma samples obtained from diabetic patients with microalbuminuria (Mic, *n* = 10), diabetes mellitus patients with normoalbuminuria (DM, *n* = 10), and normal controls (NC, *n* = 10). The samples were analyzed using the miRNA TaqMan low-density array, which contained a total of 325 miRNAs.
