# Supplementary figures and images for: Expression Profiling and Clinical Significance of Plasma MicroRNAs in Diabetic Nephropathy
Source: J Diabetes Res. 2019 May 14;2019:5204394. doi: 10.1155/2019/5204394 (PMC6536987; doi:10.1155/2019/5204394)

**Supplementary data** The complete graph of the array chip


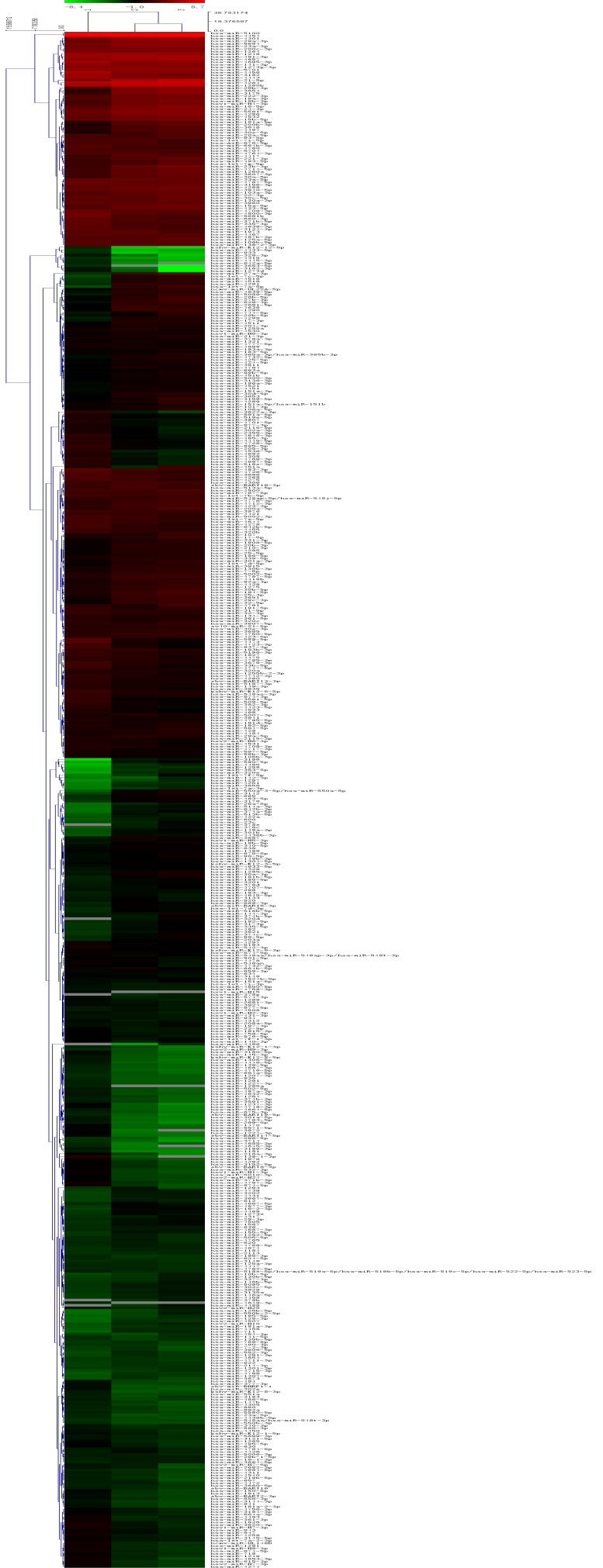

Supplement: Supplementary Materials — Supplementary Figure 1: the complete graph of the array chip. RNA was extracted from plasma samples obtained from diabetic patients with microalbuminuria (Mic, n = 10), diabetes mellitus patients with normoalbuminuria (DM, n = 10), and normal controls (NC, n = 10). The samples were analyzed using the miRNA TaqMan low-density array, which contained a total of 325 miRNAs. [file 5204394.f1.doc]
